# New Frontiers in the Phase Structure and Functional Properties of Materials

**DOI:** 10.3390/ma16134692

**Published:** 2023-06-29

**Authors:** Ligang Zhang, Yuan Yuan

**Affiliations:** 1 School of Materials Science and Engineering, Central South University, Changsha 410083, China; 2 National Engineering Research Center for Magnesium Alloys, College of Materials Science and Engineering, Chongqing University, Chongqing 400044, China

Materials’ functional properties are strongly related to their phase structures. This Special Issue on “The Phase Structure and Functional Properties of Materials” therefore aims to introduce the latest technologies being utilized to further understand the formation of phase structures and microstructures in materials and their relationship with their functional properties in order to provide support for the advanced, knowledge-based design of materials with desired functional properties.

A prediction model for the welding process of a Ti–6Al–4V titanium alloy was established by using the finite element method to evaluate the phase composition, residual stress, and deformation of the welded joints of Ti–6Al–4V sheets involved in different processes (including tungsten inert gas welding, TIG, and laser beam welding, LBW) [1]. The results show that the effect of phase transformation on residual stress is mainly in the weld area, which affects the tensile and compressive stress in the aforementioned area. The phase composition and residual stress calculated by the established model are scalar fields which can be introduced into the numerical analysis of structural fracture failure as input influence factors.

The phase relations of zirconium-based nuclear cladding materials are also contained within the scope of the featured papers. The Fe–Zr–Y system is an important part of zirconium-based nuclear coating materials, and its high temperature resistance and radiation resistance can be greatly improved following oxidation. Although the Fe–Zr–Y system has good prospects, there are still some issues that limit its applications in industry. The effective way to solve this problem is to possess a comprehensive and in-depth understanding of the phases of different components and exclude the unstable phase at high temperatures in advance. This is very important for Fe–Zr–Y system applications. Within this Special Issue, isothermal sections’ information at 1073 K and 973 K of the Fe–Zr–Y system [2] are reported which can be utilized in the nuclear industry.

The texture evolution and texture formation mechanism of Fe-0.65%Si are also contained within the scope of this Special Issue [3]. The microstructure and texture evolution of non-oriented electrical steel produced by the CSP process during recrystallization and the grain growth process were studied in detail. The formation mechanism of α* fiber texture is expected to provide a theoretical basis to further improve the magnetic properties of CSP-process non-oriented electrical steel.

This Special Issue also contains works related to Nd–Fe–B magnets [4]. The phase formation and microstructure of (Nd_1-2x_Ce_x_Y_x_)_14.5_Fe_79.3_B_6.2_ (x = 0.05, 0.10, 0.15, 0.20, 0.25) alloys have been studied experimentally. The magnetic properties of (Nd_1-2x_Ce_x_Y_x_)_14.5_Fe_79.3_B_6.2_ melt-spun ribbons were measured by a vibrating sample magnetometer (VSM). The measured results show that the remanence (B_r_) and coercivity (H_cj_) of the melt-spun ribbons decrease with the increase in Ce and Y substitutions, while the maximum magnetic energy product ((BH)_max_) of the ribbons first decreases and then increases. This shows that the magnetic properties of Nd–Fe–B ribbons with Ce and Y co-substitution can be adjusted by alloy composition and phase formation to prepare new Nd–Fe–B magnets with the benefits of low production costs and high performance.

The studies on the synthesis, optical characterizations, and solar energy applications of new Schiff base materials are presented in Ref. [5]. The results of DSC and POM investigations have indicated that all of the synthesized series are single crystals with a nematic mesophase, except for the unsubstituted member (I6c) which is non-mesomorphic. In addition, the polarizability anisotropy of the polar compact terminal and lateral groups affects the mesomeric characters of the whole molecular architecture. Moreover, compared to the unsubstituted derivatives, this property belonging to the terminal part affects the properties of the mesophase and leads to phase transition.

The Guest Editors of this Special Issue would like to give special thanks to the authors and the editorial team of *Materials* for their involvement in the collaborative and peer-review processes. We hope you enjoy reading this Special Issue.
